# Three-Dimensional Simulation of Scalp Soft Tissue Expansion Using Finite Element Method

**DOI:** 10.1155/2014/360981

**Published:** 2014-07-09

**Authors:** Qiu Guan, Xiaochen Du, Yan Shao, Lili Lin, Shengyong Chen

**Affiliations:** ^1^College of Computer Science, Zhejiang University of Technology, Hangzhou 310023, China; ^2^School of Medicine, Zhejiang University, Hangzhou 310012, China

## Abstract

Scalp soft tissue expansion is one of the key medical techniques to generate new skin tissue for correcting various abnormalities and defects of skin in plastic surgery. Therefore, it is very important to work out the appropriate approach to evaluate the increase of expanded scalp area and to predict the shape, size, number, and placement of the expander. A novel method using finite element model is proposed to solve large deformation of scalp expansion in this paper. And the procedure to implement the scalp tissue expansion with finite element method is also described in detail. The three-dimensional simulation results show that the proposed method is effective, and the analysis of simulation experiment shows that the volume and area of the expansion scalp can be accurately calculated and the quantity, location, and size of the expander can also be predicted successfully with the proposed model.

## 1. Introduction

3D imaging has been widely applied in the current plastic and reconstructive surgery. Noninvasive computed tomography (CT), magnetic resonance imaging (MRI), and three-dimensional laser scanning are increasingly used to generate tissue structural views for 3D anatomical model [1] to assess facial growth [2, 3], facial expressions [4, 5], facial asymmetry of cleft lip and palate patients [6], and facial reconstruction [7]. This kind of techniques is also expected to model the reconstruction accurately and to make plastic surgery planning as a truly interactive procedure. Many literatures have also shown that various 3D skeleton models have been made in maxillofacial surgery. During a planning process, the simulation of osteotomy can be checked by the computer-aided design (CAD), where the actual lines of osteotomy can be clearly identified with the help of CAD.

**Figure 1:**
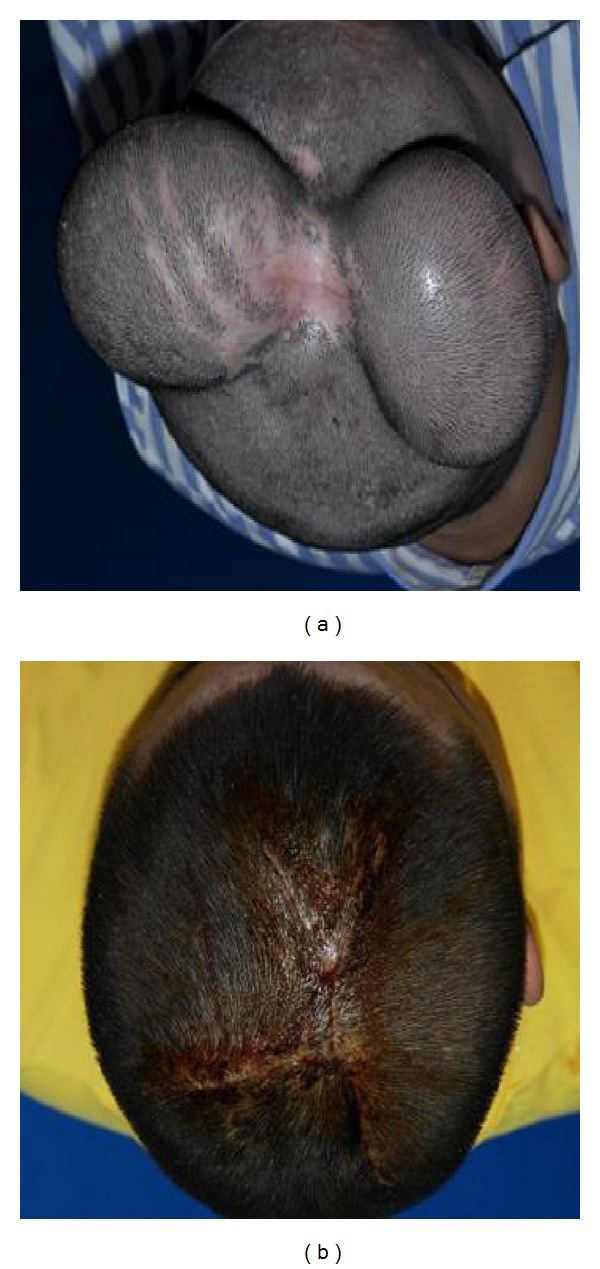
Skin soft tissue expansion surgery.

However, the assessment of soft tissue in the plastic surgery is more difficult than bones due to the biomechanical properties of soft tissue, such as nonhomogeneous, quasi-incompressible, and nonlinear plastic-viscoelastic material properties. Therefore, computer-aided based soft tissue expansion technique is introduced into three-dimensional imaging to assess the structure of soft tissue. Skin soft tissue expansion technique (as shown in Figure 1) is also one of the key medical techniques in surgery planning, which is used to generate new skin flaps for correcting various skin abnormalities and defects. Therefore, it is widely applied in many fields such as plastic and reconstructive surgery, cosmetic surgery, and reparative and reconstructive surgery. It is also very useful and practical in the treatment of soft tissue defects in the head and facial area. Meanwhile, it is critical for a successful surgery planning to effectively select the shape, size, number, and buried location of the expander according to the practical state of the defect skin. Because inadequate expanded flap size may result in failure of covering the skin defect without enough tension and overexpanded flap size means wasting tissue, Ji et al. [7] obtained the data of the scar excision in a child with burned injuries and the expanded cervicofacial flap by using a 3D digital scanner. The proposed result shows that the scar area planned for excision matches the area of the face and anterior neck with tissue expansion well. But the result is of theoretical value, and the influence of biomechanical properties of soft tissue has not been considered during the whole procedure, such as wound retraction and flap shrinkage.

So far, the accuracy of operation still mainly relies on the surgeon's clinical experience accumulated from long-term practices. It is quite subjective and unstable. To solve the problems well, simulation of tissue deformation during its expansion process can provide an additional modality to improve the mission success rate in tissue expansion surgery, so it will be discussed in detail in this paper. The main purpose of this study is to find out the appropriate approach to obtain more accurate data considering wound retraction and flap shrinkage after removing the tissue expanders in surgery, which is based on the images generated from CT scans. The CAD-based mathematical model is then to be constructed to simulate the process of the surgery, which consisted of selecting the proper expanders, removing the tissue expanders, and covering the soft tissue defect with the expanded flaps.

In Figure 1(a), there is a practical example of two liquid expanders implanted into the patient's head; what is shown in Figure 1(b) is the healed wound after removing expanders.

The rest of this paper is organized as follows.  Section 2 provides the methods employed to simulate scalp soft tissue expansion.  Section 3 focuses on the experiments and the discussion of the method and experiment results.  Section 4 presents a conclusion and the future work.

## 2. Simulation of Scalp Soft Tissue Expansion with Finite Element Method

**Figure 2:**
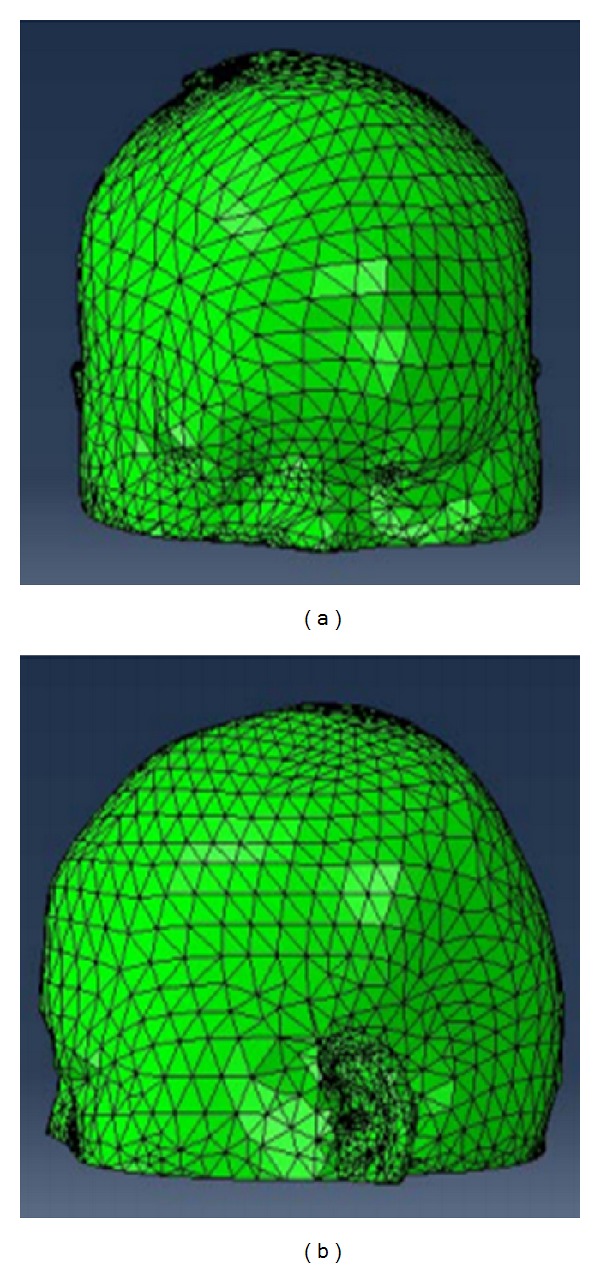
Scalp model represented by tetrahedral grids.

Geometric model is the foundation of deformable simulation. And three-dimensional model is widely used in the simulation of medical tissue deformation. The scalp of the model usually has a certain thickness. According to the structural characteristics of the scalp, the thickness is set to 3.4 mm [8]. Besides, the model of tetrahedral grids [9] is obtained by free division method [10] using software Abaqus. The result is shown in Figure 2.

Scalp expansion is a kind of deformation in large range and nonlinear problem that contains geometric and material issues [11]. In this paper, a dynamic finite element model is introduced to solve the nonlinearity of the problem [12]. The finite element theory based geometric equations and equilibrium conditions for small deformation are no longer suitable due to the geometric nonlinearity. In general, there are two main ways to describe large deformation problem, material description (Lagrange description), and space description (Euler description) [13]. At the initial time *t*_0_ = 0, the coordinate of number of *i* points is *X*_*i*_ (*i* = 1,2, 3) and becomes *x*_*i*_ after motion at any time. The motion of the point can be represented by the following equation:

(1) $$\begin{array}{c} x_{i}=x_{i}\left(X_{i},t\right),\;i=1,2,3. \end{array}$$

The above equation is the Lagrange description used in this paper that examines the movement and deformation using the motion of a specific point. The configuration of the object before deformation is already known and is also as a reference model. The configuration of the object after deformation is computed by finite element method. Currently, the pair of second class Piola-kirchhoff stress tensor and Lagrant-Green strain tensor is widely used to express the energy item using finite element method to solve nonlinear problem. This pair tensor takes initial configuration as reference configuration. The solving steps of scalp soft tissue expansion with finite element method are as follows.

### 2.1. Discrete and Equal Parameter Unit Interpolation

For the initial configuration, its geometry of internal units is interpolated with the coordinates of the unit points. Besides, unit displacement is also obtained by the same interpolation function as follows:

(2) $$\begin{array}{c} X_{i}=\overset{m}{\underset{k=1}{\sum }}N_{k}X_{i}^{k},\\ u_{i}=\overset{m}{\underset{k=1}{\sum }}N_{k}u_{i}^{k}, \end{array}$$

where *X*_*i*_^*k*^ is the coordinate of point *K* before deformation in the direction *i*, *u*_*i*_^*k*^ is the displacement of the point *k* in the direction *i*, and *m* is the number of the unit points. They can be represented as vectors, *X* = *NX*_*e*_ and *U* = *Na*_*e*_, *N* = [*N*_1_ *I*, *N*_2_ *I*,…, *N*_*m*_ *I*], where *I* is a 3 × 3 unit matrix and *N* is a 3 × 3*m* shape function matrix. Vector *X*_*e*_ is the coordinate vector of the initial unit point and *a*_*e*_ is the displacement vector of the unit point. Both of them are 3*m* in dimension.

### 2.2. Derivation of Strain Matrix *B*

Strain matrix is represented by the strain tensor of Green in this paper. At first, strain tensor of Green *E* is represented by two parts, linear part *E*_*L*_ and nonlinear part *E*_*N*_. So, there are *E* = *E*_*L*_ + *E*_*N*_ and *B* = *B*_*L*_ + *B*_*N*_. *B*_*L*_ is the transformational matrix between *E*_*L*_ and *a*_*e*_; that is, *E*_*L*_ = *B*_*L*_ *a*_*e*_. *B*_*N*_ is the transformational matrix between *E*_*N*_ and *a*_*e*_, and *E*_*N*_ = *A*∗*θ*/2. The detailed solving process of *B*_*L*_ and *B*_*N*_ is described as follows:

(3) $$\begin{array}{c} B_{L}=LN=\left[LN_{1},LN_{2},\ldots ,LN_{m}\right],\\ L=\left[\begin{array}{cccccc} \frac{\partial }{\partial X_{1}} & 0 & 0 & 0 & \frac{\partial }{\partial X_{3}} & \frac{\partial }{\partial X_{2}}\\ 0 & \frac{\partial }{\partial X_{2}} & 0 & \frac{\partial }{\partial X_{3}} & 0 & \frac{\partial }{\partial X_{1}}\\ 0 & 0 & \frac{\partial }{\partial X_{3}} & \frac{\partial }{\partial X_{2}} & \frac{\partial }{\partial X_{1}} & 0 \end{array}\right], \end{array}$$

(4) $$\begin{array}{c} A=\left[\begin{array}{cccccc} \frac{\partial U}{\partial X_{1}} & 0 & 0 & 0 & \frac{\partial U}{\partial X_{3}} & \frac{\partial U}{\partial X_{2}}\\ 0 & \frac{\partial U}{\partial X_{2}} & 0 & \frac{\partial U}{\partial X_{3}} & 0 & \frac{\partial U}{\partial X_{1}}\\ 0 & 0 & \frac{\partial U}{\partial X_{3}} & \frac{\partial U}{\partial X_{2}} & \frac{\partial U}{\partial X_{1}} & 0 \end{array}\right],\\ \theta =\left[\begin{array}{ccc} \frac{\partial U^{T}}{\partial X_{1}}, & \frac{\partial U^{T}}{\partial X_{2}}, & \frac{\partial U^{T}}{\partial X_{3}} \end{array}\right], \end{array}$$

where *θ* is a displacement gradient matrix, ∂*U*/∂*X*_*i*_ is a 3 × 1 matrix, and 0 represents a 3 × 1 zero matrix. And then *B*_*N*_ can be calculated as in the following equation. That is *B*_*N*_ = *AG*, where *G* is obtained by

(5) G=[I∂∂X1I∂∂X2I∂∂X3][N1IN2I⋯NmI]=[∂N1∂X1I∂N2∂X1I⋯∂Nm∂X1I∂N1∂X2I∂N2∂X2I⋯∂Nm∂X2I∂N1∂X3I∂N2∂X3I⋯∂Nm∂X3I].

### 2.3. System Balance Equation

For a triangle composed of three points, second class Piola-kirchhoff stress tensor can be described as $S={\left[\begin{array}{cccccc} S_{11} & S_{22} & S_{33} & S_{23} & S_{31} & S_{12} \end{array}\right]}^{T}$ . Physical force load and surface force load are, respectively, represented as $P_{0}={\left[\begin{array}{ccc} P_{01} & P_{02} & P_{03} \end{array}\right]}^{T}$ , $q_{0}={\left[\begin{array}{ccc} q_{01} & q_{02} & q_{03} \end{array}\right]}^{T}$ . System balance equation using virtual work equation is defined as

(6) $$\begin{array}{c} c^{t}\overset{}{\underset{e_{0}}{\int }}B^{T}S\;dV=c^{t}\overset{}{\underset{e_{0}}{\int }}N^{T}P_{0}\;dV+c^{t}\overset{}{\underset{A_{e_{0}}}{\int }}N^{T}q_{0}\;dA, \end{array}$$

where matrix *c* combines displacement vector *a*_*e*_ of element point with the total displacement vector *a* of finite element system via the equation of *δa*_*e*_ = *c* *δa*. The balance equation of the whole system can be defined as (7) and obtained by adding the total unit balance equations given as (6)

(7) $$\begin{array}{c} \overset{}{\underset{e_{0}}{\int }}B^{T}S\;dV=\overset{}{\underset{e_{0}}{\int }}N^{T}P_{0}\;dV+\overset{}{\underset{A_{e_{0}}}{\int }}N^{T}q_{0}\;dA. \end{array}$$

To solve (7), scalp constitutive model is needed to be introduced and defined as follows [14]:

(8) $$\begin{array}{c} W=\frac{a}{b}\left\{\exp\left[\frac{b}{2}\left(I_{1}-3\right)\right]-1\right\},\\ I_{1}=\lambda_{1}^{2}+\lambda_{2}^{2}+\lambda_{3}^{2}, \end{array}$$

where *w* represents strain energy density, *I*_1_ represents the first strain invariant, *λ*_1_, *λ*_2_, and *λ*_3_ are the stretch variables, respectively, in directions *x*, *y*, *z*, and *a*, and *b* represents parameter *s* of the expander. Because scalp constitutive model is a superelastic material model and *W* = *W*(*I*_1_, *I*_2_, *I*_3_), the scalp is defined as superelastic material in this paper. Considering that external force has nothing to do with the deformation path of the material, *I*_1_ = *E*_*ii*_, *I*_2_ = [(*E*_*ii*_)^2^ − *E*_*ij*_ × *E*_*ji*_]/2, and *E*_*ii*_ = *E*_*ij*_ = *E*_*ji*_. For incompressible material, *I*_3_ = 1. The constitutive equation with rate form can be obtained by derivation of deformation variables

(9a) $$\begin{array}{c} S_{ij}^{\prime }=\frac{\partial W}{\partial E_{ij}}=D_{ijkl}^{T}E_{kl}^{\prime }, \end{array}$$

(9b) $$\begin{array}{c} D_{ijkl}^{T}=\frac{\partial^{2}W}{\partial E_{ij}\partial E_{kl}}, \end{array}$$

where *S*_*ij*_′ is the derivation of time by second class Piola-kirchhoff stress tensor, *E*_*kl*_′ is the derivation of time by Green strain tensor, and *D*_*ij* *kl*_^*T*^ is tangent modulus tensor. For a three-dimensional model, object region is *V*_0_ and boundary is *A*_0*t*_. The equivalent nodal load *R* of FEM system can be gotten by combining *D*_*ij* *kl*_^*T*^ with balance equation as described in the following equation:

(10) $$\begin{array}{c} R=\overset{}{\underset{v_{0}}{\int }}N^{T}P_{0}\;dV+\overset{}{\underset{A_{0t}}{\int }}N^{T}q_{0}\;dA=a\overset{}{\underset{V_{0}}{\int }}B^{T}DB\;dV. \end{array}$$

### 2.4. System Tangent Stiffness Matrix *K*_*T*_

In general, it is difficult to obtain accurate stiffness matrix for nonlinear material, so stiffness matrix can be replaced by tangent stiffness matrix which is the curve tangent of stress-strain defined as follows:

(11) $$\begin{array}{c} \overset{}{\underset{v_{0}}{\int }}B^{T}S\;dV+\overset{}{\underset{v_{0}}{\int }}{\left(dB\right)}^{T}S\;dV=K_{T}da, \end{array}$$

where *K*_*T*_ = *K*_*M*_ + *K*_*S*_ and *K*_*M*_ = *K*_*L*_ + *K*_*N*_. *K*_*M*_ is a tangent stiffness matrix associated with constitutive matrix, *K*_*L*_ is a general small displacement stiffness matrix, *K*_*N*_ only including a linear or quadratic term is caused by a large displacement, and *K*_*S*_ is a tangent stiffness matrix by stress

(12) $$\begin{array}{c} K_{L}=\overset{}{\underset{V_{0}}{\int }}{B_{L}^{T}D}_{T}B_{L}\;dV,\\ K_{N}=\overset{}{\underset{V_{0}}{\int }}\left(B_{L}^{T}D_{T}B_{N}+B_{N}^{T}D_{T}B_{N}+B_{N}^{T}D_{T}B_{L}\right)dV. \end{array}$$

*D*_*T*_ in formula (12) contacts unit stress tensor and strain tensor, representing *dS* = *D*_*T*_ *dE*. *K*_*S*_ can be calculated by (13)

(13) $$\begin{array}{c} K_{S}=\overset{}{\underset{V_{0}}{\int }}{\tilde{G}}^{T}\tilde{M}\tilde{G}\;dV, \end{array}$$

where $\tilde{G}$ is the transformation matrix of displacement gradient vector and unit nodal displacement vector.

### 2.5. Solving Equations by Newton Method

**Step 1 .**

Solving linear elastic problem *K*_*L*_ *a* − *R* = 0, first approximate solution *a* ^1^ is obtained. *a* is the total displacement vector of FEM system.

**Step 2 .**

Compute matrix *A* from *a* ^1^ based on (4). Displacement gradient vector *θ* = *Ga*_*e*_. Compute *E* through *δAθ* = *A*(*δθ*) based on the definitions of *A* and *θ*. Then, compute *S* ^1^ and *B* ^1^ based on scalp constitutive model *D* and *S* = *DE*

**Step 3 .**

Get unbalanced force *φ* via the left part of (9a) minus the right part of (9b). Get *φ* ^1^ through *S* ^1^ and *B* ^1^.

**Step 4 .**

Solve tangent stiffness matrix *K*_*T*_^1^ with (11) to (13).

**Step 5 .**

Compute the correction of displacement via Δ*a* ^1^ = −(*K*_*T*_^1^)^−1^ *φ* ^1^. Get second approximate solution *a* ^2^ = *a* ^1^ + Δ*a* ^1^.

**Step 6 .**

Iteration on *a* ^2^ from Step 2 to Step 5 till *φ* ^*n*^ is small enough.

### 2.6. Conditions of Loading and Boundary

The expander usually is set near the defect area of patient. The stress of the scalp packaging expander is caused by liquid in expander and the direction of stress is along the normal expander's surface. Therefore, the stress of scalp contact parts is the same as the stress of expander, which is set as 30 N/cm^2^ in this paper. Besides, the expander is set as constant regular ellipsoid whose triaxial proportion is 1.53 : 1 : 1. Suppose the volume of the virtual ellipsoid is zero and the center point of the virtual ellipsoid is at the center of specified area at the beginning. During the scalp deformation, the ellipsoid expands and its volume increases. The distance between the center point and scalp surface is computed to decide the virtual ellipsoid and scalp surface whether the virtual ellipsoid and the scalp surface is contacted or not.

## 3. Experiments Result and Analysis

### 3.1. Experimental Result

**Figure 3:**
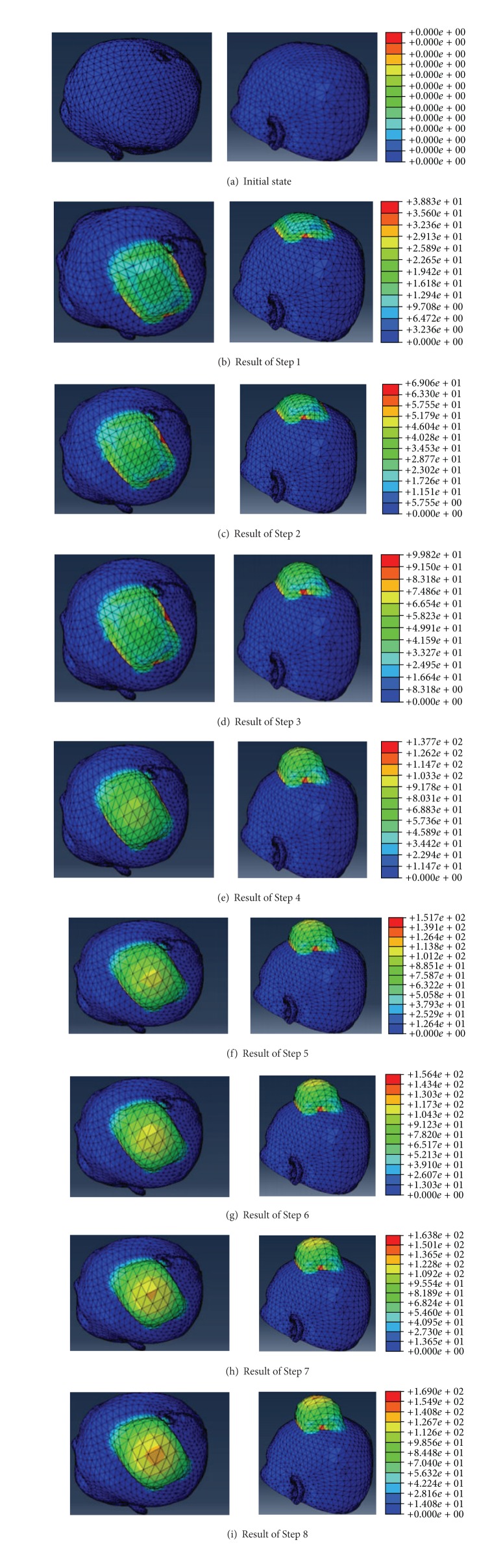
Results of scalp expansion simulation.

The simulation is based on the large deformation of scalp expansion and conditions of loading and boundary above. Eight steps are set in software Abaqus and the time of each step is 0.04 s. Initial state and main process results of the 8 steps are shown in Figure 3.

### 3.2. Stress-Strain Analysis

**Figure 4:**
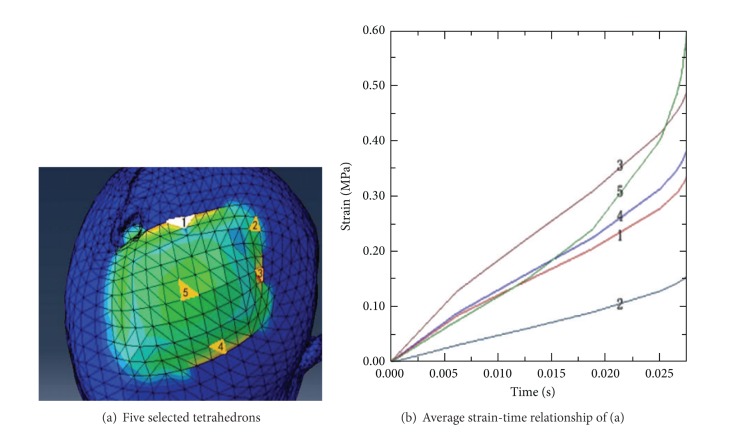
Stress-strain analysis diagram.

The color in Figure 3 represents the average stress distribution: red means the maximum and blue means minimum. It is obvious that the stress is relatively large in confined areas and the stress decreases from the top of scalp to the bottom of scalp. Five tetrahedrons are selected and the average strain-time relationship of four points of each tetrahedron is shown in Figure 4.

As shown in Figure 4, the stress of tetrahedron 1, 3, or 4 at the edge of area is larger than that of tetrahedron 5 from 0 s to 0.016 s. When the expander is implanted into head, the downward stress from scalp is large, the strain of top tetrahedron 5 is restricted, and tetrahedrons 1, 2, 3, and 4 around expand outward. Then, when the expansion strain of the edge regional is growing and the volume of expander is increasing, the expansion to the top of scalp becomes obvious. Therefore, the strain of tetrahedron 5 increases faster than that of tetrahedrons 1, 2, 3, and 4. With the restrictions of the fixed scalp around, the strain of tetrahedrons 1 and 2 is quite small. The strain of tetrahedron 3 is relatively large for the corner position. The strain of tetrahedron 4 is neither large nor small for the middle layer position.

### 3.3. Area Analysis

**Figure 5:**
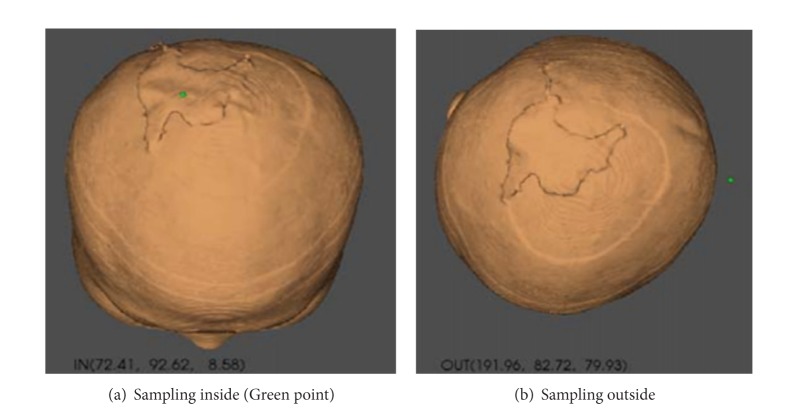
Random sampling inside and outside the model.

**Figure 6:**
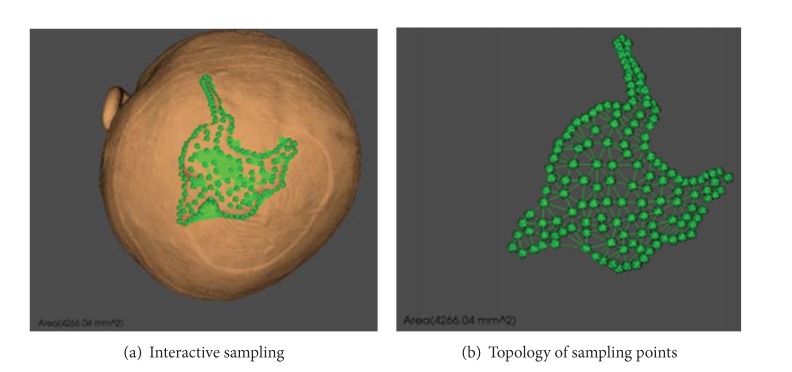
Getting area by interactive sampling in real time.

Before the expansion, coordinates of scalp surface model are obtained at random. Then, these points are connected in delta in real time using Delaunay triangulation method [15] and the area of each triangle is computed. After accumulation, the area of defect is 4266.04 mm^2^ at last. The process is shown in Figures 5 and 6.

With the process of calculation result by software Abaqus, the area of scalp after deformation is shown in Table 1.

After removing the expander, skin shrinkage phenomenon will occur. The part of shrinkage will be 30% based on medical experience, so about 1.43 times more new skin is needed. After the end of the experiment, the area is increased to 6789.54 mm^2^ which is 1.59 times of the defective area (4266.04 mm^2^). Considering the utilizable efficiency of new skin, the surgery can be satisfied.

### 3.4. Volumetric Analysis

In order to estimate the volume needed in the operation of scalp tissue expansion, each step of deformation is processed by software and the volume is shown in Table 2.

As Table 2 shows, the volume of head increases totally 442012.12 mm^3^ (442.01212 mL). It is almost equal to the volume of expander indeed. So, a 450 mL ellipsoid expander is needed to be implanted. The results show that the large deformation method proposed in this paper is effective.

## 4. Conclusion

Tissue expansion is a good option for covering the soft tissue defect. Successful reconstruction is depended on the precise judgement on the amount of tissue provided by expansion to cover the defect. The three-dimensional anatomy alters the situation that elasticity and contractility of the expanded flap make it extremely difficult to accurately predict the proper size implants and the size of skin flaps required to cover the defects.

Based on small deformation and linear elastic problems with finite element method, a novel solution to large deformation of scalp expansion is put forward in this paper. Then, the concrete steps to implement the scalp tissue expansion process with finite element method are also given in detail. The scalp tissue is simulated as a shell with certain thickness and is split into tetrahedral meshes. The deformation results prove that the solution for large deformation is effective. Then, the stress during the deformation process is also analyzed, and the volume and the area of the scalp are accurately calculated. With the proposed method, the quantity, placement location, quantity, and size are predicted successfully.

However, the proposed model used to approximate flap shrinkage is rough, and it does not take into consideration other variables such as thickness of flaps and the length of expansion time which will make the rate of shrinkage different. Further studies are required to make the model more accurate.

## Figures and Tables

**Table 1:** The area of scalp after deformation.

Step	*S* (mm^2^)	Δ*S* (mm^2^)	∑Δ*S* (mm^2^)
0	117733.11	0	0
1	118504.79	771.68	771.68
2	119319.77	814.98	1586.66
3	120392.57	1072.8	2659.46
4	122278.13	1855.56	4545.02
5	123252.50	974.37	5519.39
6	123638.54	386.04	5905.43
7	124240.53	601.99	6507.42
8	124522.65	282.12	6798.54

**Table 2:** The change of volume.

Step	*V* (mm^3^)	Δ*V* (mm^3^)	∑Δ*V* (mm^3^)
0	3107136.50	0	0
1	3166015.68	58879.18	58879.18
2	3220374.85	54359.17	113238.35
3	3289162.78	68787.93	182026.28
4	3404764.16	115601.38	297627.66
5	3467026.40	62262.24	359889.9
6	3491925.98	24899.58	384789.48
7	3530934.93	39008.95	423798.43
8	3549148.62	18213.69	442012.12
